# Pivoting Dental Practice Management during the COVID-19 Pandemic—A Systematic Review

**DOI:** 10.3390/medicina56120644

**Published:** 2020-11-25

**Authors:** Syed Sarosh Mahdi, Zohaib Ahmed, Raheel Allana, Alessandro Peretti, Francesco Amenta, Mohammed Nadeem Bijle, Liang Lin Seow, Umer Daood

**Affiliations:** 1 Department of Community Dentistry, Faculty of Dentistry, Jinnah Medical and Dental College, Sohail University, Karachi 74800, Pakistan; 2 Centre of Clinical Research, Telemedicine and Tele Pharmacy, School of Medicinal and Health Products Sciences, University of Camerino, 62032 Camerino, Italy; alessandro.peretti@unicam.it (A.P.); francesco.amenta@unicam.it (F.A.); 3 College of Dental Medicine, Columbia University, New York, NY 10027, USA; dr.zohaib@live.com; 4 Department of Paediatrics and Child Health, Aga Khan University Hospital, Karachi 74800, Pakistan; dr.raheelallana@hotmail.com; 5 Paediatric Dentistry, Faculty of Dentistry, The University of Hong Kong, Hong Kong 999077, China; mnbijle@connect.hku.hk; 6 Division of Clinical Dentistry, School of Dentistry, International Medical University Kuala Lumpur, 126, Jalan Jalil Perkasa 19, Bukit Jalil, Wilayah Persekutuan, Kuala Lumpur 57000, Malaysia; lianglin_seow@imu.edu.my (L.L.S.); umerdaood@imu.edu.my (U.D.)

**Keywords:** COVID-19, pandemic, simulation, dentistry, oral health, infection control, dental practice, management

## Abstract

*Background and Objectives*: The aims of this systematic review were to identify additional infection control measures implemented in dental practice globally to prevent cross-infection and evaluate the psychological impacts of the pandemic among dental professionals. *Materials and Methods*: A sequential systematic literature search was conducted from December 2019 to 30 April 2020 through PubMed, CINAHL, Scopus, Google Scholar, Embase, and Web of Science databases. The search yielded the following results: “COVID-19” (*n* = 12,137), “Novel corona virus” (*n* = 63), “COVID-19 and dentistry” (*n* = 46), “COVID-19 and oral health” (*n* = 41), “Novel Corona virus and Dentistry” (*n* = 0), “dental health and Novel Coronavirus” (*n* = 26), and “dental practice and Novel Coronavirus” (*n* = 6). Results: After a careful review and eliminating articles based on inclusion and exclusion criteria, the final review included 13 articles. Management of infection control is discussed extensively in the literature and remains the main theme of many Coronavirus Disease 2019 (COVID-19) articles on dentistry. Telephone triage using a questionnaire, hand hygiene, personal protective equipment (PPE) for clinical and nonclinical staff, a preprocedural mouth rinse, and aerosol management have been discussed and implemented in few countries. Three studies recommended that elective treatments for patients with a temperature of >100.4 F or 38 °C should be postponed or performed in an airborne infection isolation room (AIIR) or negative-pressure room. Limiting the number of patients in the waiting area, the removal of shared objects, proper ventilation, and physical distancing were highly recommended. Psychological distress among dental professionals in relation to existing medical conditions and self-efficacy has been discussed. *Conclusions:* Although the COVID-19 pandemic has had a substantial impact on the dental profession worldwide, our review highlights many practice management approaches to adopt the new norm. More research highlighting evidence-based safety practices and multisectoral collaboration is required to help dental professionals make informed decisions and make the profession safe, both for the patient and dental professionals.

## 1. Introduction

Coronavirus Disease 2019 (COVID-19), a pneumonia of unknown origin, was reported in Wuhan, a town in Eastern China. The World Health Organization office in China provided the first reports of a new virus of unknown origin. A surveillance system was implemented, and patients’ throat swabs were sent to laboratories for etiological analysis [1]. The causative agent was identified by the Chinese Center for Disease Control and Prevention, and the causative Wuhan seafood market was shut down immediately [2]. Initially, the virus was named the 2019 novel coronavirus; later, it was named Severe Acute Respiratory Syndrome Coronavirus 2 (SARS-CoV-2), as suggested by the Coronavirus Study Group (CSG) of the International Committee on the Taxonomy of Viruses [3]. On 30 January 2020, the World Health Organization (WHO) declared the rapid spread of SARS-CoV-2 and its associated Coronavirus Disease 2019 (COVID-19) as a public health emergency of international concern with an overall case-fatality rate of 3.4% [4,5]. Precisely 34,838,209 confirmed COVID-19 cases and 1,033,356 deaths have been reported globally (as of 3 October 2020) [6].

Scientific studies have shown that there are two main routes of transmission of COVID-19: direct (person-to-person touch or inhalation of short-range respiratory droplets) and indirect (airborne and fomite-mediated) [7,8,9]. Individuals may acquire indirect infection by getting into contact with contaminated surfaces and touching their oral, nasal, or ocular mucosal surfaces [10,11]. The infection is spread by large droplets formed by symptomatic patients while coughing and sneezing, which may also occur in infected, asymptomatic individuals [9,11,12,13]. Many symptoms have been reported, but the most commonly cited clinical symptoms are raised body temperature, dry cough, malaise, and dyspnea [12]. Computerized Tomography (CT) scan findings included pneumonia with anomalous findings in all cases. Although all demographics of the global population are at risk for COVID-19 infection, healthcare workers and patients hospitalized for other reasons are at a higher risk for COVID-19 due to the possibility of frequent close contact with symptomatic and/or asymptomatic individuals infected with COVID-19 [14].

**Figure 1:**
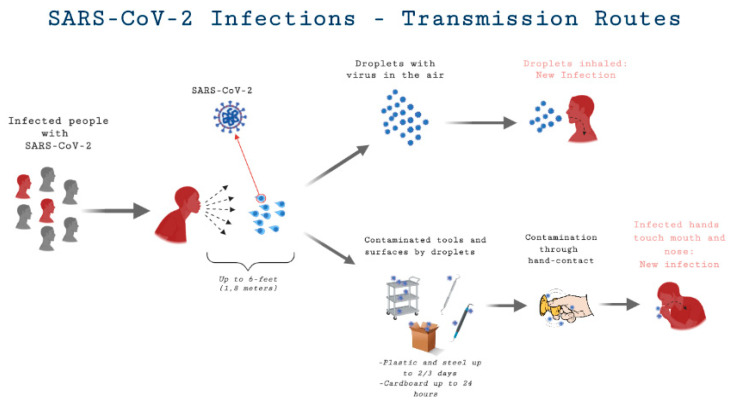
Coronavirus Disease 2019 (COVID-19) transmission routes.

The dental fraternity is experiencing a drastic transition and will continue to do so over the coming weeks and months due to the SARS-CoV-2 outbreak [15]. The consequences are far-reaching and unpredictable, particularly for the dental community and patients seeking dental care. A study from the National Institute of Health (NIH) found that aerosols containing SARS-CoV-2 remain infectious for up to 3 h in confined spaces, 4 h on copper, 24 h on cardboard, and up to 3 days on stainless steel and plastic [16]. Figure 1 demonstrates the possible route of transmission for SARS-Cov-2 in dental practice. The use of an ultrasonic scaler, a triple syringe, dental handpiece, and other high-speed-driven instruments during dental treatment can generate significant amounts of aerosols, putting dental professionals, dental teams, and their patients at high risk for contracting COVID-19 [16,17,18,19]. In the initial phases of the pandemic, there were many recommendations in the United States and elsewhere to cease nonessential dental procedures and restrict treatment to emergency care. However, with additional measures and protocols in place, many dental practices have been able to provide routine healthcare delivery during the pandemic [20,21,22,23].

Dental practitioners are facing uncertainty and being forced to rely on general information of COVID-19 transmission routes and other recommendations being followed by general frontline health staff to protect themselves and their patients [21]. With its outbreak, COVID-19 has raised the bar for taking additional measures along with universal infection control standard precautions.

Therefore, the aims of this systematic review were (i) to identify additional infection control measures implemented in dental practice globally to prevent cross-infection, and (ii) evaluate the psychological impact of the pandemic among dental professionals.

## 2. Materials and Methods

Two independent reviewers (S.S.M. and Z.A.) screened the titles and abstracts of all the identified studies to determine their relevance and whether they met the predetermined inclusion criteria (Table 1). The authors screened PubMed, Scopus, Web of Science, Embase, CINAHL, and Google Scholar databases from December 2019 to 30 April 2020 for appropriate articles addressing the question under review. The researchers carefully reviewed all the articles based on the desired outcome(s) by reading the abstracts of the relevant publications. Full texts were screened if there was insufficient detail to make a clear decision based on the inclusion criteria. A structured and logical approach to the literature search was used to identify the relevant papers that investigated COVID-19 and dental practice implications. Reference lists of original studies were manually searched to identify any articles that could have been missed during the initial search, keeping the inclusion criteria in mind. Any disagreements regarding study selection were resolved via discussion (Table 1 of inclusion and exclusion criteria).

### 2.1. Outcome and Review Question

The primary outcome was “dental practice management”, particularly the management of infection control procedures through additional measures during the COVID-19 pandemic. The secondary outcome was psychological distress among dental professionals during the COVID-19 pandemic.

Based on the Preferred Reporting Items for Systematic Review and Meta-Analysis (PRISMA) guidelines, a specific question was constructed [24]. The addressed question in focus was “What additional measures have been taken around the globe for the management of potentially infectious persons visiting a dental practice?”

### 2.2. Data Sources and Study Selection

**Figure 2:**
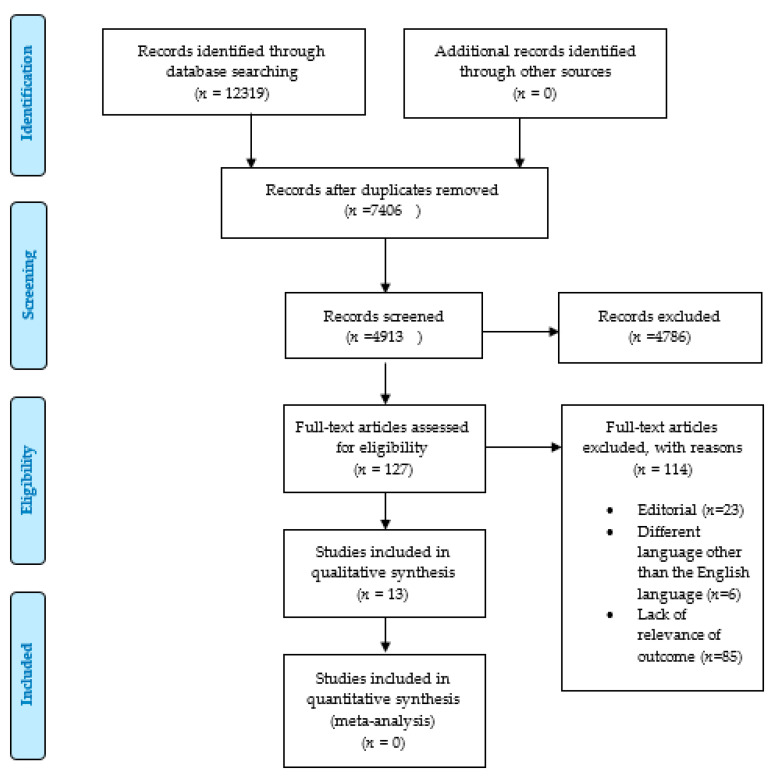
Preferred Reporting Items for Systematic Review and Meta-Analysis (PRISMA) flow chart.

A sequential systematic literature search was conducted on the above-mentioned databases using the following keywords: “COVID-19” (*n* = 12,137), “Novel corona virus” (*n* = 63), “COVID-19 and dentistry” (*n* = 46), “COVID-19 and oral health” (*n* = 41), “Novel Corona virus and Dentistry” (*n* = 0), “dental health and Novel Coronavirus” (*n* = 26), and “dental practice and Novel Coronavirus” (*n* = 6) (Table 2). A Medical Subjects heading (MeSH) search strategy did not return any articles, probably due to the novelty and scarcity of articles for the topic under review. MeSH terms “Coronavirus and Dentistry,” “Corona virus and oral health,” “COVID-19 and dentistry,” and “COVID-19 and oral health” were used, which gave zero results (PRISMA flow chart, Figure 2). The search parameters used for the inclusion criteria were articles written in the English language, with at least one keyword corresponding to the above entries in the title/abstract, and studies based on the evaluation of research articles (Table 3). Original articles and systematic reviews addressing COVID-19 and dentistry were entitled to inclusion. Correspondences and editorials were not included. All keyword searches, title and abstract screenings, as well as the selection of studies, were carried out independently by two investigators (Drs. S.S.M./R.A.). Articles published before December 2019 were not included, as the subject of novel coronavirus only emerged into the scientific conscience and mainstream after December 2019. Furthermore, online outpatient department (OPD) reports/patient–physician testimonials and other online materials were skimmed for other relevant material (PRISMA flowchart, Figure 2). Given the heterogeneous and emerging sources relevant to dental aspects of COVID-19, only articles published in peer-reviewed journals were selected for final review to provide dental professionals with the highest level of evidence. We were able to gather a considerable number of studies that could serve as the initial groundwork for providing strategies to successfully manage safety in dental practice. Endnote 8.0 was used to organize the extracted references. The majority of the studies we used in the final sample are original studies and discussions based mainly on original studies; however, we included some reviews due to the dearth of articles on the topic and that, for reviewing on a scientific basis, one may evaluate other secondary or systematic review papers. Review articles were used to identify original articles on the topic of interest, and some of the conclusions of the various studies were used in the discussion. COVID-19 in dentistry has frequently been the topic of reviews in the last few months, and our project required a few review articles to build a complete scientific picture. As such, there was a crossover of some parallel review studies. This is obviously a very novel topic and an ongoing event; therefore, only original articles were evaluated for quality assessment through the Newcastle–Ottawa Scale.

Exclusion Criteria: Studies or other materials published before December 2019 were not included in our analysis. Therefore, the selected articles were published between January 2020 and April 2020. Papers not published in peer-reviewed journals were excluded, as well as studies not matching the inclusion criteria and desired outcomes. Case reports and case series were also excluded along with editorials. Articles published in any language other than English were discarded as none of the authors were fluent in other languages.

### 2.3. Risk of Bias Assessment

Assessment of the risk of bias of the reviewed studies is a fundamental aspect of conducting a systematic review. Bias risk assessment in the reviewed studies was examined using the Newcastle–Ottawa scale (NOS) [25]. The NOS scale can only be applied to original studies; therefore, only original articles were graded using the NOS scale. The Newcastle–Ottawa scale is a tool for quality assessment and ranking studies by assigning them stars (*), and a modified version was used in the study, which used a 10-star rating system instead of the usual nine. The stars are ranked by 3 key domains of the study being assessed (selection, comparability, and outcome). The NOS can be interchanged with the commonly used Agency for Healthcare Research and Quality Standard Assessment (ARHQ). The more stars, the lesser the risk of bias in the studies included. Each study is rated as poor (0–4 *), fair (5–6 *), or good (7–9 *). The results of the assessment are displayed in this article (Table 3).

**Table 3:** Newcastle–Ottawa scale quality assessment form for nonrandomized studies included in the review.

	Selection					Comparability		Outcome			Overall
Study & Year	1	2	3	4	5	6	7	8	9	10	Score (*)
Kamate S.T et al., 2020 [26]	*	*	*			*		*		*	6
Schacham et al., 2020 [27]	*	*		*		*		*	*	*	7
Ahmed M.A et al., 2020 [28]	*	*		*	*	*	*			*	7
Khader Y et al., 2020 [29]	*	*			*	*		*		*	6
Yang Y et al., 2020 [30]	*	*	*			*	*	*	*	*	8
Al Harbi et al., 2020 [31]	*	*			*	*		*	*		6

(*) = poor (0–4 *), fair (5–6 *), or good (7–9 *).

### 2.4. Statistical Analysis

For the evaluation of reliability, inter and intra observer reproducibility were evaluated using the weighted Kappa (κ_w_) statistic using IBM SPSS for Windows version 22.0 (IBM Corp., Armonk, NY, USA).

## 3. Results

A total of 12,319 articles were extracted for screening (Figure 2). The removal of duplicates resulted in 4913 articles. An additional 4786 articles were excluded based on title and abstract, leaving 127 for full-text assessments. The final systematic review included 13 publications after excluding 114 articles, which comprised editorial letters, lacked relevance to our desired outcomes, or used languages other than English.

Another search was done following the same keywords from May to 30 September. It produced 1066 articles, out of which only six articles demonstrated our objective question. Out of six articles, three assessed knowledge, attitude, and awareness of dentists towards the COVID-19 pandemic [32,33,34,35]. One study assessed the risk of aerosol transmission in a dental setup [19], and another demonstrated the psychological impact of dentists towards COVID-19 [36]. Moreover, the findings were consistent with the articles published between December 2019 and 30 April 2020.

To this date (30 April 2020), six original articles and seven review articles were identified and extracted. Of 13 studies, nine addressed the primary outcome of practice management, particularly infection control management [30,31,37,38,39,40,41,42,43]. The remaining four discussed psychological distress, phobia, awareness, and self-efficacy during the COVID-19 pandemic among dental professionals [26,27,28,29]. With the majority of shortlisted publications from China [30,37,38,39,43] discussing additional infection control measures along with standard precautions, one each was conducted in Austria [40], the USA [41], and Italy [42]. A telephone triage to screen out suspected COVID-19 cases using a questionnaire was implemented in several studies [31,38,39,41,42]. Three studies recommended that elective treatments for patients coming with a temperature >100.4 F or 38 °C should be postponed if possible or performed in an airborne infection isolation room (AIIR) or negative-pressure room [31,39,41]. Minimal invasive procedures as an alternative to aerosol-generating procedures are recommended, such as the use of CariSolv for caries removal [37], extraoral radiographs [41], which are preferred over intraoral radiographs to prevent gag, and the use of a hand scaler where a rubber dam is unavailable [31]. Likewise, a two-before-and-three-after hand hygiene guideline recommended by the CDC (Centre for Disease Control) and WHO has been suggested [26,37,39,41,42]. An extraoral high-volume suction for aerosol-generating procedures [37,39], a pre-procedural mouth rinse [31,37,38,39,41,42], limiting the number of patients and displaying cough etiquette in the waiting area [39] with proper ventilation, and physical distancing were extra measures implemented to safely manage dental practice during the pandemic [30,33,36,41,43]. The theme of protective masks was recurrent in the literature, and differing views were observed. Some authors suggested wearing an FFP1/standard surgical mask for non-aerosol-generating procedures and FFP2/N95 or higher for aerosol-generating procedures [37,39]. Several others suggested using FFP2/N95 for all procedures for both clinical and nonclinical staff (clinicians and assistants) [41,42,43]. Waste management and the psychological impact of COVID-19 on the global dental workforce was another theme explored extensively in the literature [26,28,29,37,42,43].

Among four studies evaluating the psychological impact of the COVID-19 pandemic using cross-sectional surveys among dental professionals [26,27,28,29], it was observed that the majority of dental providers across the globe were nervous and frightened by the disastrous consequences of the pandemic. One of the studies highlighted the possible interaction between psychological distress in relation to self-efficacy and a pre-existing medical condition among dental professionals [27]. Dental professionals with a lower score for self-efficacy and any pre-existing medical conditions had elevated psychological distress, and vice versa [27]. A summary of the characteristics of the reviewed articles is presented in Table 4. The weighted Kappa for intraobserver reproducibility exceeded the 0.70 cut off, with a mean of 0.86, indicating almost a perfect reproducibility, while the mean weighted Kappa (κ_w_) for interobserver reproducibility was 0.80, showing substantial reproducibility.

## 4. Discussion

According to local and regional health authorities’ recommendations during the COVID-19 pandemic, the protective measures that should be undertaken in a dental setting can be categorized into four phases: (a) patient triage, (b) patient evaluation upon arrival (c) during dental treatment, and (d) after dental treatment.

### 4.1. (a) Patient Triage Prior to Patient Arrival

Patient triage for the detection of suspected/confirmed cases of COVID-19 and determining the need for emergency and urgent dental care are among the policies and procedures that can be considered prior to the patient’s arrival at the dental clinic. Six out of the thirteen articles from different geographical locations (including China, USA, Italy) and practice settings implemented telephone triage using a questionnaire to evaluate the potential risk of SARS-Cov-2 transmission and type of dental care needed [31,37,38,39,41,42]. Telephone triage using a screening questionnaire was implemented. Emergency dental care for patients who reported symptoms of COVID-19, had contact with COVID-19-infected individuals, or traveled to regions with a high number of COVID-19 cases in the past 14 days was postponed for two weeks, and pharmacologic management of pain or infection was considered [36,41,44]. More recently, a new protocol was adopted to delay any treatment for patients with confirmed cases of COVID-19 until two consecutive negative swab tests were established taken 24 h apart [44]. A few articles mentioned that the same questionnaire should be repeated, and body temperature should be documented using a noncontact thermometer upon the patient’s arrival at the clinic [37,41,42].

### 4.2. (b) Patient Evaluation and Screening Upon Arrival

Screening of patients, maintaining a 1 m physical distance in the dental office, use of face masks for those accessing the dental office, patient education, and use of personal protective equipment (PPE) by dental staff are among the activities to be carried out in the dental office. The patient must be unaccompanied in the treatment room and any caregiver should remain in the waiting area [22,44]. Dental professionals should also consider reducing the number of patients in the waiting area and increasing the amount of time for each visit to complete the maximum possible treatment in one visit to reduce repeated exposure [19,33,36]. Upon arrival, the patient should be asked to disinfect their hands with an alcohol-based sanitizer [36,45]. Patients with a temperature of >100.4 F or 38 °C requiring urgent dental care should have their appointments postponed if possible or performed in an airborne infection isolation room (AIIR) or negative-pressure room [31,39,41]. These are single-patient isolated rooms with a minimum of six air changes per hour [39,46]. Air from these rooms is exhausted outside, away from areas of human traffic or gatherings. It is filtered through a high-efficiency particulate air (HEPA) filter with a negative-pressure monitoring system held in place [39,46,47]. More recent recommendations provided by healthcare authorities emphasize that patients in the waiting area must wear a mask, gloves, and eye protection [36,41,44,48]. Ge et al. suggested displaying cough etiquette instructions at the entrance and in the waiting area to promote respiratory hygiene, which should also include reminders for physical distancing and wearing a mask all the time [39].

### 4.3. (c) Infection Control during Dental Treatment

Ensuring hand hygiene, providing preoperative antimicrobial mouth rinse to patients, utilizing rubber dams and high-volume saliva ejectors, minimizing aerosol-generating procedures and extraoral radiographs, a one visit treatment, and disinfection operations should be carried out throughout dental procedures. Before starting the dental procedure, a preprocedural mouth rinse for 60 s with an oxidizing agent such as 1% hydrogen peroxide or 0.2% povidone–iodine to reduce the viral load in aerosols has been suggested [11,37,41,42,49]. Several studies reported that chlorhexidine might not be effective against SARS-Cov-2 because there is a lack of evidence and systemic data, and the virus is primarily susceptible to oxidation [37,39,42,44]. The use of a rubber dam and high-volume evacuation/suction (HVE) during aerosol-generating restorative procedures has been suggested to reduce both airborne and surface contamination [11,18,31,37,41,42,50,51,52]. A rubber dam could potentially provide a 70% reduction in aerosols and eliminate all sources of aerosol contamination from blood or saliva by blocking the throat and soft tissue area, except the tooth/teeth undergoing treatment [50,51,52]. High-volume evacuator (HVE) or expensive high-efficiency particulate arrestor (HEPA) filters, if held within 6–15 mm of the aerosol-generating tip, can clean up to 90% and 99.99% of contaminated air, respectively [39]. Peng et al. also recommended a minimally invasive chemomechanical agent, CariSolv, for the removal of carious dentine, in addition to a hand scaler for periodontal procedures where a rubber dam is not feasible [19,37]. Although there is emerging evidence on the effectiveness of an antiretraction valve for eliminating the risk of cross-infection, its use has been suggested as an additional preventive measure to reduce cross-contamination and the dispersion of droplets or aerosols [11,37,53].

There has been much debate about the choice of a filtering face-piece (FFP), level 1 vs. level 2 vs. level 3, for aerosol- and non-aerosol-generating dental procedures. Some authors suggested wearing an FFP1/standard surgical mask for non-aerosol-generating procedures and FFP2/N95 or higher for aerosol-generating procedures [37,39]. Others suggested FFP2/N95 for all procedures for clinicians, dental surgery assistants, and front desk staff [41,42,43]. A systematic review of clinical trials assessing the effectiveness of N95 respirators in comparison to a standard surgical mask found no additional protection in preventing influenza [52]. Evidence from SARS-CoV research suggests that small infectious particles of up to 3 μm remain airborne indefinitely in an isolated room [54]. Patients and dental professionals can be exposed via inhalation of sustained small airborne infectious particles upon entering a room that was used to perform aerosol-generating procedures when wearing minimal airborne protection and using a standard surgical mask [18,37,43]. Therefore, considering the highly infectious nature of SARS-Cov-2 compared to influenza, we recommend the use of FFP2/N95 for both clinical and nonclinical staff for all dental procedures. Due to the risk of transmission from asymptomatic individuals and those in an incubation period, every patient should be considered potentially contagious [13,18].

Hand hygiene using a two-before-and-three-after technique recommended by the CDC and WHO has been extensively emphasized as a key factor in preventing cross-contamination [26,37,39,41,42]. In China, dental professionals were advised to disinfect their hands with soap or 70–90% alcohol before examination, before procedure, after touching the patient, patient-contaminated surroundings or instruments, and exposure to bodily fluids [37,39]. Reusable eye protection with safety glasses and a face shield were implemented in Italy and China [37,42]. Alharbi et al. recommended the use of extraoral radiographs such as orthopantomogram and cone-beam computer tomography over intraoral radiographs to prevent gagging and excessive salivation [31]. Overall, a layering approach, including head covers, long-sleeved water-resistant gowns, shoe covers, level 2 FFP, and eye protection for both clinical and nonclinical staff, has been suggested [37,39,41,42].

### 4.4. (d) Disinfection after Treatment

Disinfection of the treatment room and waiting area, including doorknobs, chairs, floor, desks, restrooms, and elevators between patients, has been suggested [39,41,42]. Hospital-grade disinfectants, including quaternary ammonium-based, phenol-based, and alcohol-based products such as 0.1% sodium hypochlorite or 70% isopropyl alcohol, have proven to be effective against coronaviruses [11,55,56]. The literature suggests the use of UVC light, a HEPA filter air purifier, or room ventilation for 30 min prior to surface disinfection after treatment may reduce the risk of infection [57,58]. However, we only found few articles from Italy and China where treatment/waiting area ventilation was implemented between patients [33,36,39,42].

Cleaning and disinfection of reusable facial protective devices and the handling of surgical waste after normal procedures should be considered after dental care. Waste management was another theme explored extensively in the literature. The treatment and disposal of medical waste pose indirect health risks due to environmental contamination; therefore, medical waste should be disposed of in accordance with the protocols provided by local health authorities. Peng et al. suggested that a temporary storage area should be assigned in the clinic for the storage of medical waste [37]. Reusable instruments should be adequately pretreated using an oxidizing disinfectant, cleaned, sterilized, and stored in accordance with the local health authorities’ protocol [11,37]. Additionally, double-layered packing, appropriate labeling, and gooseneck ligation have been suggested for medical waste generated from suspected/confirmed cases of COVID-19 [11,37]. Contaminated disposable PPE, including gloves, gowns, and head covers, should be safely disposed of in a bag within the clinical area before entering nonclinical areas.

### 4.5. Psychological Impact of COVID-19 among Dental Professionals

Four articles examined awareness, perception, attitudes, and behavior among dental professionals regarding the COVID-19 pandemic [26,27,28,29]. Dental healthcare professionals are at high risk for acquiring and transmitting the infection to their peers, families, and other patients due to the possibility of exposure to suspected/confirmed COVID-19 patients [18,28]. This is especially important as emotional instability due to fear and anxiety can foster irrational behavior and inadequate infection control practices [28]. Khader et al. conducted a cross-sectional study among 368 Jordanian dentists from different clinical settings to assess awareness, perception, and attitude regarding COVID-19 and infection control practices [29]. Jordanian dentists were found to have limited knowledge about the incubation period for the viral infection, physical distancing, masks for patients in the waiting area, hand hygiene practices, and protective clothing for clinical and nonclinical staff. Over 80% reported avoiding treatment for suspected/confirmed COVID-19 cases amid fear of contracting the disease [29]. Another study conducted by Ahmed et al. surveying 669 dentists from 30 different countries reported that almost 80% feared contracting COVID-19 and indicated avoiding treating suspected cases [28]. This is further backed by scientific evidence available from previous research showing an unwillingness of dental providers to treat patients with infectious diseases such as SARS, HIV, tuberculosis, and MERS [27,28]. The use of a rubber dam and preprocedural mouth rinse with an oxidizing agent were ignored by the majority of dental providers [28]. A high level of anxiety was reported among dental professionals related to practice closure and subsequent economic implications [28]. A study evaluating psychological stress experienced by Israeli dentists and dental hygienists during the COVID-19 pandemic identified that elevated psychological distress was significantly associated with having an existing chronic medical condition, a low self-efficacy score, and contracting COVID-19 from patients [27]. The study further highlights that psychological distress among dental professionals may have long-term effects and recommends mental health education or workshops to enhance self-efficacy [27].

Other studies assessing knowledge, awareness, and the psychological impact of COVID-19 among dental professionals from India [32], Italy [33,36], and Poland [34] have reported high perceived risk and a low level of awareness concerning infection management. A cross-sectional survey assessing hygiene practices among Indian dental professionals found that 33% were unaware of adequate use of PPE and only 60% were aware of guidelines recommended by the WHO [32]. One-third of the participants did not disinfect the lab work area, and almost 40% had a low knowledge score measured on a Likert scale [32]. Dental professionals with postgraduate education had a significantly high level of knowledge compared to graduates [32]. Another study from Italy assessing the psychological impact of COVID-19 among dental professionals using the General Anxiety Disorder-7 (GAD-7) test found an overall mild level of anxiety in 33% of the participants due to the pandemic [36]. The anxiety level was significantly positively correlated with concerns about the professional future, contracting the COVID-19 disease, and the risk of infection transmission among patients [36]. Using a self-reported questionnaire, the study also identified that 1% of dental practitioners contracted COVID-19 and 70% knew at least one person (friend/patient/colleague) who tested positive [36]. Continuing educational (CE) courses can benefit dental providers by providing them with infection control protocols in dental settings during the COVID-19 pandemic. However, a survey from Northern Italy found that only 30% of dental providers completed a CE course on COVID-19, where only 2% claimed to be confident in avoiding infection [36]. Other factors affecting dental professionals’ attitudes include adequate access to PPE, continuity of clinical practice, and type of practice (public compared to private) [34]. Another survey of Italian dental practitioners assessing the perceived risk of aerosol contamination during the COVID-19 pandemic found that the majority (70%) of dentists believed fine-aerosol-producing treatments to be of increased risk [19]. A more recent study related to disinfection knowledge, attitude, and practices among dental professionals during the COVID-19 pandemic globally found that almost 50% failed to correctly indicate surface disinfectant as an effective measure against COVID-19 [35]. The study also found inconsistent knowledge regarding the stability of SARS-CoV-2 on different surfaces, and the majority of dental providers believed chlorhexidine to be an effective preprocedural mouth rinse against SARS-CoV-2 [35].

The role of local authorities in providing procedural guidelines in the face of the pandemic is vital to help healthcare providers in making informed decisions. As identified in this review, adequate knowledge of the incubation period, routes of transmission, adequate use of PPE, and disinfection protocols are essential to curb the chain of transmission and safely treat suspected/confirmed COVID-19 patients [19,29,32,33,34,35,36].

### 4.6. Limitations

This review has few limitations pertaining to ongoing emergency due to the COVID-19 pandemic and substantial heterogeneity in the selection of sources addressing infection control management in the dental practice. Additionally, cross-sectional surveys addressing psychological distress among dental professionals are subjective/self-reported and may present reporting.

## 5. Conclusions

The COVID-19 pandemic presents a substantial risk for dental professionals and the community. Hence, only strict adherence to protocols can protect dental teams and patients from contracting COVID-19. The future trajectory of COVID-19, strength of individual healthcare systems, availability of rapid testing kits, vaccines, and successful therapeutic options for COVID-19 are factors that could possibly influence dental practice and additional precautionary measures that dental practitioners should adopt in the coming weeks and months. More research is required on aerosol’s specific risk assessment and measures that can protect the dental workforce and patients from aerosol and droplet infection. The economic and psychological aspect of the COVID-19 pandemic also needs special attention as the pandemic is taking a toll on the mental health of large segments of the population in these unprecedented and stressful times. It is important to fill in the gaps in knowledge regarding the complex nature of COVID-19′s impact on dental practice.

## Figures and Tables

**Table 1:** Inclusion and exclusion criteria.

Inclusion Criteria	Exclusion Criteria
Articles published after the COVID-19 outbreak (Dec 2019 onwards)	Studies published prior to COVID-19
English language only	Articles not in the English language
Original articles and reviews	Editorials, opinions, perspective, correspondences, case reports, case series
Only articles published in peer-review and indexed journals	Non-peer-reviewed/nonindexed journals
Databases examined (PubMed, CINAHL, Scopus, Embase, Google Scholar)	Little or no focus on dental aspects

**Table 2:** Keywords and search strings.

Keywords/Search Strings	Number of Entries Found
COVID-19	12,137
COVID-19 and dentistry	46
Novel corona virus	63
COVID-19 and oral health	41
Dental practice and novel corona virus	6
Dental health and novel corona virus	26
Novel corona virus and dentistry	0

**Table 4:** Descriptive characteristics of articles reviewed.

First Author (Date)	Type of Article	Population/Setting	Article Key Points	Recommendations/Outcomes	Limitations
Peng, et al., 2020 [31]	Review Article	Setup: Routes of 2019-nCoV transmission and control in dental practice. Context: China	Dental care environments typically bear the possibility of contamination with 2019-nCoV owing to the complexity of its practice, which requires face-to-face contact with patients. Patients and dentists can be exposed via inhalation of suspended airborne particles, indirect contact from contaminated surfaces, and direct contact with bodily fluids of infected individuals.	In-clinic patient evaluation using a noncontact thermometer for fever.	Lack of evidence for the effectiveness of chlorhexidine mouth rinse against 2019-nCoV
				A two-before-and-three-after hand hygiene guideline.	
				Standard protection for nonclinical staff (gloves, surgical mask, cap, goggles, and white coat).	
				Advanced protection for clinical staff (standard protection with an extra layer and shoe cover).	
				Tertiary protection with special protective outwears for contact with 2019-nCoV-infected patients.	
				A preprocedural mouth rinse with 1% hydrogen peroxide or 0.2% povidone.	
				Rubber dam and extra-high-volume suction. Use of a hand scaler for periodontal scaling and CariSolv for caries removal where a rubber dam is unavailable. Antiretraction valve handpieces.	
				Gooseneck ligation is suggested for the disposal of medical waste.	
Meng, L. et al., 2020 [32]	Review Article	Setup: Risk of cross-infection in dental settings. Context: China.	This study offers critical information regarding COVID-19 and nosocomial infections in dental settings. Management and guidelines for dental practitioners and students in (potentially) infected areas.	Stringent personal safety precautions.	None
				Precheck triages to document temperature for staff and patients.	
				Online lectures for students.	
Ge, Z. et al., 2020 [33]	Review Article	Setup: Transmission and control of COVID-19 infection in dental settings via aerosol. Context: China	The article emphasizes the importance of aerosol transmission of COVID-19 in dental settings and the implementation of precautionary measures to limit its spread.	Cough etiquette instructions in the waiting area.	Distinguishing symptoms of fever and fatigue caused by dental infections from COVID-19 is merely dependent on the practitioner’s expertise. Effect of preprocedural mouth rinse against SARS-Cov-2 with oxidative agents and chlorhexidine is still unknown.
				Multidisciplinary consultation and negative-pressure rooms for management of suspected/confirmed cases of COVID-19.	
				Mechanical ventilation in treatment and waiting area, 70–90% alcohol for hand hygiene using the two-before-three-after technique; level 1 facemask for non-aerosol-generating procedures level 2 face mask for aerosol-generating procedures, and level 3 face mask for confirmed/suspected case of COVID-19.	
				Rubber dam for aerosol-generating procedures, and 60 s surface disinfection of waiting and treatment areas. HEPA or high-volume evacuator (HVE) within 6–15 mm from an aerosol-generating tip can clean up to 99% or 90% of contaminated air, respectively.	
Kamate, S.K. et al., 2020 [40]	KAP Study	Setup: 860 dental practitioners from different continents. Context: Global	The present research was conducted with the intention of evaluating the awareness, behaviors, and activities (KAP) of dental practitioners in light of the COVID-2019 pandemic.	Dentists were found to have good knowledge and practice scores.	A bias in social desirability.
				All dentists accepted that they should help spread knowledge of the disease, and that hand hygiene and PPE were successful in avoiding COVID-19.	Owing to the cross-sectional aspect of the sample and the sampling method used, the effect of self-selection of the part of the respondents may have arisen. Questionnaire bias.
Yang, Y. et al., 2020 [34]	Original Article	Setup: 48 public tertiary dental hospitals. Context: China	The state of nonemergency dental care, emergency dental facilities, online consultation, and regional spread of hospitals were evaluated during the COVID-19 pandemic.	All 48 public tertiary dental hospitals discontinued regular nonemergency dental care and were offering emergency dental care only.	Within the limitation of this report, we observed significant changes in the health service provision of Chinese public tertiary hospitals during the COVID-19 epidemic. Nonetheless, more research should concentrate on the possible long-term effects that the outbreak may contribute on dental treatment.
				The penetration rate of teleconsultation in the eastern area was considerably higher compared to central and western regions.	
Zimerman et al., 2020 [35]	Review Article	Setup: Risk of COVID 19 risk in Oral and Maxillofacial Surgery Department. Context: Vienna	The purpose of the study is therefore to compile and address facets of the treatment of patients in oral and maxillofacial surgery during the COVID-19 pandemic.	Correct usage of personal protection equipment.	Necessary investments should be made for dreadful future situations.
				All patients should be considered infectious.	
				Outpatient visits should be reduced to a minimum.	
				All patients who are admitted to the inpatient unit should undergo a standard SARS-CoV-2 examination.	
Ather, A. et al., 2020 [36]	Review Article	Setup: Recommendations for clinical dental practice in COVID-19. Context: USA	The purpose of this article is to provide a summary of the epidemiology, symptoms, and mechanisms of transmission of this novel infection. Implications for clinical dental practice in response to COVID-19 have been highlighted.	Every patient should be considered infectious. Telescreening and triaging to identify suspected COVID-19 patients.	Likelihood of treating an asymptomatic COVID-19 patient in a dental setting is high due to the large incubation period from 0 to 24 days and mild presentation of disease in some individuals.
				Defer elective dental care for 2 weeks for patients with fever (>100.4 F = 38 °C).	
				Six feet distance and surgical masks for patients in a well-ventilated waiting area.	
				Pharmacological management of urgent care for suspected or confirmed COVID-19 cases.	
				Follow CDC guidelines for reuse of N95 to preserve PPE.	
				Preoperative mouth rinse with 0.2% povidone–iodine or 0.5–1% hydrogen peroxide to reduce viral load, use of disposable instruments, and blood pressure cuff to prevent cross-contamination; extraoral radiograph preferred over intraoral if warranted, followed by the use of a double barrier over the sensor, rubber dam to prevent splatter, limiting the use of ultrasonic/high-speed hand piece/3-way syringe, negative-pressure treatment rooms/airborne infection isolation rooms (AIIRs) for suspected or confirmed cases.	
AlHarbi, A. et al., 2020 [37]	Original Article	Setup: Recommended provisions for dental care during the COVID-19 pandemic. Context: Global	This research sought to establish recommendations for the treatment of dental patients before and after the COVID-19 pandemic.	The recommendations for the delivery of dental treatment during the COVID-19 pandemic were established after analysis of the severity of the COVID-19 pandemic and were focused on grouping patients according to symptoms and requirements and assessing treatments according to risk and benefit.	The recommendations established in this research are general guidance and the final decision will always be made at the discretion of the practitioner.
				In addition to standard infection control measures telescreening, medical clearance for recovered cases, airborne infection isolation rooms (AIIRs) or negative-pressure rooms, extraoral radiograph over intraoral to prevent gag reflex, preoperative use of 0.23% povidone–iodine for 15 s, disposable instruments and devices, rubber dam, minimally invasive procedures, and reducing aerosol-generating procedures has been recommended.	
				Use of ibuprofen for pain management should be avoided in suspected or confirmed cases of COVID-19.	
Khader, Y. et al., 2020 [41]	Cross-sectional study	Setup: 368 Jordanian dentists from private clinics, hospitals, and health centers. Context: Jordan	The study assessed the degree of understanding, interpretation, and attitude of COVID-19 and infection management among Jordanian dentists.	However, most dentists were conscious of COVID-19 symptoms, transmission routes, and standard infection control protocols but had limited understanding of additional safety measures to prevent the spread of COVID-19 infection between patients and staff.	Low response rate, Selection bias, and sampling error limits the generalizability of the findings
Izzeti, R. et al., 2020 [38]	Review Article	Setup: Risk of transmission of COVID-19 in dental practice and preventive measures. Context: Italy.	There is a substantial risk of direct and indirect transmission of COVID-19 among dental practitioners and between patients when performing dental procedures with handpiece under irrigation due to generation of aerosol and surface/environmental contamination.	Double-phase triage telephonic followed by in-clinic to identify high-risk patients; pre- and postoperative handwashing for 60 s followed by application for 60% hydroalcoholic solution; pre- and postoperative mouth rinse for the patient with oxidative agents for 1 min; level 2/3 facemask for nonclinical staff; level 2/3 facemask, long-sleeved water-resistant gowns, surgical glasses, surgical cap, shoe cover for clinical staff; limiting the use of ultrasonic/handpiece instrument and overall treatment time; use of a rubber dam and surgical aspiration; 5 min ventilation of surgical and waiting room between patient; and surface disinfection with 0.1% sodium hypochlorite or 70% isopropyl alcohol has been suggested for the prevention of COVID-19 transmission in dental settings.	There is a lack of systemic data on the use of chlorhexidine against SARS-CoV-2. Lack of evidence, data, and unpredictable nature of this disease is affecting the adequate delivery of clinical dental care.
Xu, R. et al., 2020 [39]	Review Article	Setup: Role of Saliva in transmission and diagnostic tool for 2019-nCoV. Context: China	The article discusses saliva being a potential noninvasive diagnostic tool for 2019-nCoV detection and a potential transient medium for the spread of infection via short-distance droplets or sustained airborne aerosols.	Wearing masks, disinfecting indoor air, and maintaining social distance can prevent the dissemination of infectious salivary droplets.	None
Ahmed, et al., 2020 [42]	Cross-sectional Study	Setup: 669 dentists from 30 different countries. Context: Global	The present research measured distress and fear of infection among dentists operating during the current viral epidemic. In addition, the dentist’s information on various practice modifications in the battle against a novel coronavirus disease epidemic has been analyzed.	More than two-thirds of general dental practitioners (78%) from 30 countries were nervous and frightened by the disastrous consequences of COVID-19.	Information gathered over a concise span of time, keeping in mind the sudden effect this epidemic had on the mindset and dental professionals. Responses from all countries impacted by the outbreak were not received. Owing to the cross-sectional design of the research, we were unable to establish a cause-and-effect connection.
				A significant majority of dentists (90%) were conscious of recent improvements to care protocols.	
Shacham, M. et al., 2020 [43]	Cross-sectional study	Setup: 338 Israeli dentists. Context: Israel.	The analysis examined the correlation of COVID-19 variables and psychological factors with psychological distress in dental workers during the outbreak of the COVID-19 pandemic.	As far as self-efficacy is concerned, our findings indicate that dental workers with a higher score for self-efficacy have shown lower psychological suffering.	Cross-section model, which precludes causal inferences. Low response rate. Selection bias and sampling error.
				With respect to observational evidence, our studies have shown that dentists who have a history of disease have reported elevated clinical distress.	
